# Analysis of anatomical characteristics of congenital pulmonary airway malformation lesions based on CT images

**DOI:** 10.3389/fped.2025.1576380

**Published:** 2025-05-23

**Authors:** Yongxuan Peng, Yuejuan Xu, Jiazhong Tang, Ziling Qian, Jie Hu, Wei Liu, Yulian Xia, Xin Sun, Kun Sun, Kai Bai, Yanan Lu

**Affiliations:** ^1^ Department of Pediatric Heart Center, Xinhua Hospital, School of Medicine, Shanghai Jiao Tong University, Shanghai, China; ^2^ Department of Pediatric Cardiology, Xinhua Hospital, School of Medicine, Shanghai Jiao Tong University; Engineering Center for Congenital Heart Disease Device Diagnosis and Treatment, Ministry of Education, Shanghai, China; ^3^ Shanghai Fourth Rehabilitation Hospital (Public Welfare Hospital), Shanghai, China; ^4^ Clinical Research Unit, Xin Hua Hospital Affiliated to Shanghai Jiao Tong University School of Medicine, Shanghai, China

**Keywords:** congenital pulmonary airway malformation, pulmonary vascular segmentation, 3D slicer, CT, lobectomy (lung)

## Abstract

**Objective:**

The surgical treatment of congenital pulmonary airway malformations (CPAM) remains a subject of debate. This study aimed to deeply analyze the preoperative CT imaging data to explore the anatomical characteristics of CPAM lesions, providing additional information to guide surgical treatment for such conditions.

**Methods:**

A retrospective analysis was conducted on the medical records of children with congenital pulmonary airway malformations who underwent surgery between 2020 and 2024 at our hospital. Preoperative CT images were processed using 3D Slicer software to analyze the volume, external boundaries, vascular and airway branching, and resection planes (lung venous branch planes) of the lesions. The primary analysis indicators included lesion volume, the volume ratio of the lesion to the affected lung lobe, and the real airway, artery, and venous branches supplying the lesion, as well as the airway, artery, and venous branches that might be severed during resection.

**Results:**

A total of 17 cases and 18 corresponding preoperative CT images were included, with 7 cases using enhanced CT scans. The mean age of the patients was 68.9 ± 38.9 months. Most lesions (72.2%) were located in the lower lung. The average volume of the lesions was 47.5 cm^3^ (range: 25.6–91.4 cm^3^), which occupied 26.6 ± 12.7% of the affected lung lobe. There was no significant correlation between lesion volume and age (*r* = 0.25), and a weak negative correlation between the volume ratio and age (*r* = −0.48). The proportion of lesions with real supplying airway, artery, and venous branches was 16.7%, 77.8%, and 83.3%, respectively. The proportions of lesions requiring the severance of additional airway, artery, and venous branches during resection were 27.8%, 16.7%, and 5.5%, respectively. The external boundaries of the lesions were most clearly exposed.

**Conclusion:**

The use of preoperative CT imaging and corresponding image processing software allows for a comprehensive analysis of the anatomical characteristics of congenital pulmonary airway malformation lesions. This may help improve the understanding of CPAM and the effectiveness of lesion resection surgeries.

## 1 Introduction

Congenital Pulmonary Airway Malformation (CPAM) is the most common congenital malformation of the lungs in children, with an incidence rate of 1.21 cases per 10,000 live births in China (1). CPAM generally refers to cystic or adenomatoid lesions, and its pathogenesis remains unclear, potentially due to the distal atresia of bronchi during embryonic development and various gene mutations (2). There is a standardized process for prenatal diagnosis and postnatal management of CPAM. Typically, a prenatal ultrasound detects cystic lung lesions, raising suspicion of CPAM, which is then further confirmed through fetal MRI. Postnatal follow-up focuses on chest CT scans to evaluate the anatomical characteristics of the lesion and the presence of related clinical symptoms such as recurrent respiratory infections or pneumothorax (3). CPAM requires differential diagnosis from bronchopulmonary sequestration, bronchogenic cysts, and congenital lobar emphysema, although the surgical approaches for these conditions are largely similar. Clear indications for surgical intervention include massive lesions causing respiratory and circulatory instability or recurrent clinical symptoms, while there is still some controversy regarding the indications, timing, and surgical methods for asymptomatic CPAM children. The argument for surgery primarily revolves around the potential for CPAM lesions to undergo malignant transformation, pulmonary infections, and respiratory symptoms, coupled with the effectiveness of surgery and the absence of severe complications such as impaired lung function. The argument for conservative treatment is based on the relatively low risk of malignancy and infection, and the possibility that surgery may not reduce the risk of infection or could lead to additional complications (4–8). Regarding the timing of surgery, the optimal age for surgery varies among different centers, with the main rationale for early surgery being that operating before infection or symptom onset aids in postoperative recovery (9–16).

There are also differing views on the choice of surgical methods. Video-assisted thoracoscopic surgery has gradually replaced open thoracotomy in most cases (17–19). In terms of the extent of lung tissue resection, lobectomy remains the mainstream procedure, offering advantages in terms of surgical difficulty and the risk of lesion residue. Some studies also support that lobectomy does not affect early and mid-term lung function in children (14, 20–22). Lesion resection, mainly referring to anatomic segmentectomy, is another option (23). A newer type of lesion resection, anatomic lesion resection or non-anatomic segmentectomy, involves complete resection of the lesion along its external boundary and the pulmonary vein boundary, preserving nearly all normal lung tissue (24–26). Compared to lobectomy, lesion resection carries risks such as increased surgical time, more intraoperative bleeding, lesion residue, and higher postoperative complication rates, with insufficient clinical evidence to support a clear advantage in protecting near and long-term lung function (27, 28). CPAM lesions are not always confined to a single lung segment, so anatomic segmentectomy carries the risk of lesion residue (13, 29). Anatomic lesion resection, which involves complete resection of the lesion based on its boundary without being limited by lung segment anatomy, faces uncertainties in determining this boundary and the risk of lesion residue, leading many surgeons to avoid this method. Moreover, anatomic lesion resection is more challenging, has a longer learning curve, and lacks mid- to long-term clinical follow-up results. While there are numerous clinical reports of this procedure in China, there are fewer reports abroad.

The advantages and disadvantages of lesion resection are quite pronounced, and the surgical outcomes are closely related to surgical experience (28, 30). We believe that if the surgical difficulty and the risk of lesion residue in lesion resection can be reduced, more surgeons may opt for lesion resection, including anatomic lesion resection. Therefore, we attempt to use preoperative chest CT images and medical imaging processing software to perform 3D reconstruction of the lesions, fully identifying the size, boundaries, tracheal and vascular branching, and other anatomical characteristics of the lesions preoperatively to assist surgeons in better completing the surgery.

## 2 Materials and methods

### 2.1 Inclusion criteria

A retrospective analysis was conducted on the medical records of children who underwent surgery for congenital pulmonary airway malformation (CPAM) at our center from January 1, 2020, to December 31, 2024. The inclusion criteria were as follows: (1) The child was preoperatively diagnosed with congenital pulmonary airway malformation or intralobar pulmonary sequestration (surgical strategy was the same as for congenital pulmonary airway malformation); (2) The child had preoperative CT imaging data obtained at this center; (3) Successful reconstruction of the lesion, pulmonary arteries, pulmonary veins, and trachea using preoperative CT imaging.

### 2.2 CT image quality classification

We classified CT quality based on the method described in the Expert Consensus Document of the Society of Cardiovascular Computed Tomography (31). This method categorized CT quality as Good (diagnostic), Fair (diagnostic), and Poor (non-diagnostic). However, we adapted this standard by shifting the focus from diagnostic capability to the ability to identify and reconstruct CPAM lesions. CT scan quality was classified into three levels—high, medium, and low—based on its suitability for image processing and reconstruction. High-quality CT images had to meet the following three conditions: (1) the interlobar fissure had to be clearly identifiable in ≥80% of the slice planes, (2) there was no exudation, atelectasis, or other lesions in the lung, and (3) there was no interference from respiratory artifacts. Medium-quality CT images had to meet the following three conditions: (1) the interlobar fissure had to be clearly identifiable in 50%–80% of the slice planes, (2) the lung might have had exudation, atelectasis, or respiratory artifacts, but these did not affect the segmentation or reconstruction of the lesion or the arteries and veins. Low-quality CT images met one of the following conditions: (1) the interlobar fissure could not be clearly identified in ≥50% of the slice planes, or (2) there were lesions or artifacts that affected the segmentation or reconstruction of the lesion and arteries/veins.

### 2.3 3D reconstruction and segmentation

3D Slicer software (version 5.6.2) and its embedded modules were used for semi-automatic segmentation and 3D reconstruction of the lung lobes, pulmonary vessels, airways, and lesions. The specific methods and corresponding parameters were shown in Table 1.

**Table 1:** Main methods for manual labeling and segmentation of lesions.

Anatomical structure	Main function module	CT value	Main process	Manual labeling
Lung Lobe	Interactive lobe segmentation, Paint, Erase	≤−350	Place marker points on the interlobar fissure in the coronal plane image for semi-automatic segmentation of the lung lobes	Manually correct the semi-automatic segmentation results if unsatisfactory
Airway	Interactive lobe segmentation, Paint, Erase	≤−350	Automatically segment the airway while segmenting the lung lobes	Manually improve segmentation of the lobes and segmental bronchi
Lung Vasculature	Threshold, Smoothing, Islands, Logical operator, Scissors	≥−350	First, reconstruct the full lung model with CT values ≤ −350, then use the Smoothing function (Closing, fill holes, Kernel size 4 mm) to fill the model and apply logical subtraction to obtain the lung vasculature model with CT values > −350	Small mixed structures may remain, requiring manual removal
Pulmonary Artery Pulmonary Vein	Grow from seeds, Islands, Logical operator, Scissors	≥−350	Manually label the main trunks and branches of the left and right pulmonary arteries(veins), label branches intermittently, mark the positions adjacent to arteries and veins, then use the Grow from seeds algorithm with the lung vasculature as a template to generate distal branches	There may be missing parts of the pulmonary arteries or veins, or errors in distal branch recognition, requiring manual correction; manually segment the branches of the pulmonary artery
Lesion	Threshold, Paint, Erase	The variation is large, generally at the lower limit of the lung lobe CT value	Select the threshold with the least overlap between the lesion and normal lung tissue to reconstruct the lesion	The lesion may overlap with normal lung tissue or be incompletely reconstructed, requiring manual correction
Excision Plane	Scissors	/	Lower the opacity of the lung lobe from 1 to 0.4 to reveal the internal lesion, then use the Scissors tool to excise the normal lung tissue	The excision plane should be based on the plane formed by the pulmonary vein branches, with the lesion entirely located on the excision side of the plane

### 2.4 Anatomical characteristics definitions

The anatomical characteristics of the congenital pulmonary airway malformation lesions were described using four indicators: lesion volume and volume ratio, lesion external boundary, lesion tracheal and vascular branches, and lesion venous branches. Their definitions were as follows:

#### 2.4.1 Lesion volume (volume) and lesion volume ratio (volume ratio)

**Figure 1:**
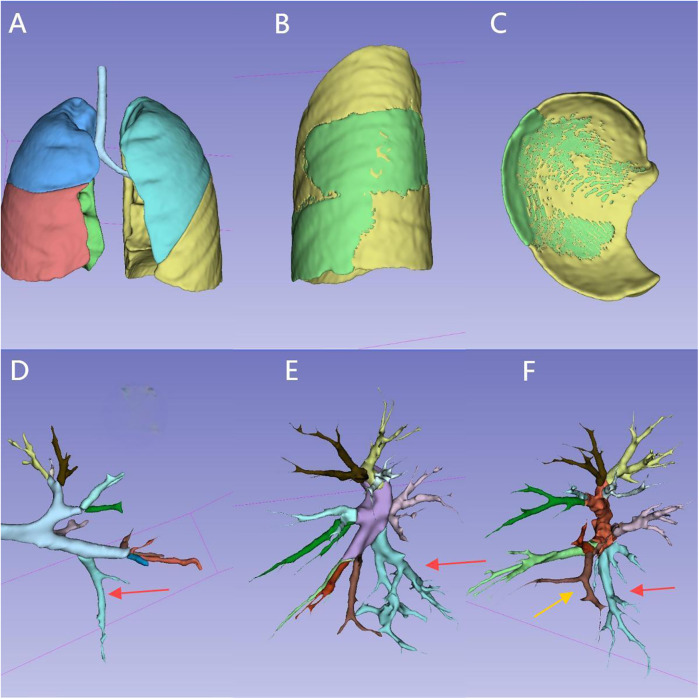
Reconstruction of the entire lung, lesion, and tracheal/vascular branches for the same case: **(A)** overall appearance of the whole lung reconstruction. **(B)** Appearance of the lesion (in the left lower lung), external boundary.(The green area represents the lesion). **(C)** Appearance of the lesion, lower boundary. **(D)** Left main bronchus and branches. (The red arrow indicates B10, the tracheal branch of the lesion). **(E)** Left pulmonary artery and branches. (The red arrow indicates A10, the arterial branch of the lesion). **(F)** Left pulmonary vein and branches. (The red arrow indicates V10, the yellow arrow indicates V9, two vein branches of the lesion). The tracheal and vascular branches of the corresponding 10 lung segments are distinguished by 10 different colors.

The lesion volume was automatically measured by the software after 3D reconstruction. The volume ratio represented the percentage of the lesion's volume relative to the total volume of the affected lung lobe (normal lung tissue + lesion tissue) (Figure 1).

#### 2.4.2 Lesion external boundary

The surface of each lung lobe was divided into three areas: lateral surface, superior surface, and inferior surface. The lateral surface corresponded to the rib-side of the lung lobe. The superior surface corresponded to the pulmonary hilum side in the upper lung, the interlobar fissure surface in the middle lung, and the interlobar fissure surface between the upper and lower lung in the lower lung. The inferior surface corresponded to the interlobar fissure surface between the middle and lower lung in the upper lung, the interlobar fissure surface between the lower and middle lung in the middle lung, and the diaphragm surface in the lower lung. The exposure proportion of the lesion's external boundary was categorized into three levels: ≥80% as high, 50%–80% as medium, and ≤50% as low. During lesion resection, the removal range was determined based on the external boundary outline, and the higher the exposure proportion, the closer it was to the actual boundary of the lesion (Figure 1).

#### 2.4.3 Lesion tracheal and vascular branches

The lesion's tracheal and vascular branches were divided into two categories: one category was the branches entering the lesion's parenchyma, defined as real branches; the second category was the branches that might need to be severed during lesion resection, defined as severed branches. Since the boundaries of lesions were often irregular, resection could not be performed precisely along the actual lesion boundary during surgery, but rather along a more continuous and smooth contour, leading to the potential severance of additional adjacent vascular branches.

#### 2.4.4 Resection plane (pulmonary vein branch plane)

**Figure 2:**
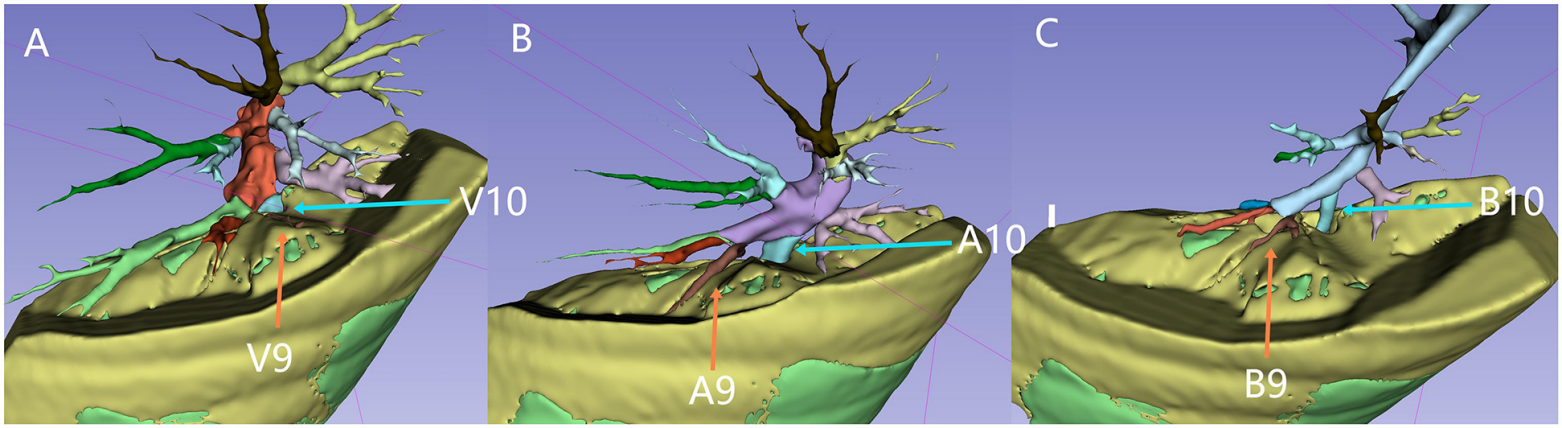
Vascular branches, tracheal branches, and the resection plane: the green area represents the lesion tissue. The yellow top surface indicates the resection plane, which is defined by an intrinsic grid plane formed by pulmonary vein branches as the internal boundary. The entire lesion is located below or at the level of this resection plane. Figures **A** to **C** illustrate the distribution of the pulmonary veins, pulmonary arteries, and left lung bronchial branches above the resection plane (on the hilar side, which needs to be preserved). The blue arrows indicate V10, A10, and B10, while the orange arrows indicate V9, A9, and B9. V9 is not visible above the cutting plane, indicating that V9, V10, A10, and B10 are branches extending into the lesion.

During image reconstruction, a virtual resection plane was constructed using the pulmonary vein branch plane near the lesion's hilum side as the marker. Based on this plane, the distribution of the tracheal and vascular branches on both the hilum side and the lesion side was analyzed to determine which specific branches would need to be severed (Figure 2, Supplementary Video S1).

### 2.5 Statistical analysis

For the numerical variables in this study, normality tests were conducted. Descriptive statistics were reported as mean ± standard deviation (for normally distributed data) or as median (interquartile range) (for non-normally distributed data). For categorical variables, frequency and percentage were calculated. Correlation analysis was performed using Pearson's Correlation Coefficient (for normally distributed data) or Spearman's Rank Correlation (for non-normally distributed data). This study did not involve comparisons of statistical differences.

## 3 Results

### 3.1 Baseline characteristics of study participants

A total of 27 children underwent 29 surgeries. Excluding 4 children who underwent CT scans at external hospitals, the remaining children underwent preoperative chest CT scans, resulting in 25 preoperative CT images from 23 children (GE MEDICAL SYSTEMS, with scanning parameters and methods following standard protocols).Of the 25 CT scan images, 5 were excluded from reconstruction and segmentation due to low image quality(1 case had multiple lesions in the right lower and upper lung lobes, and 1 case involved postoperative pneumothorax with reoperation for lung rupture repair), 2 cases were simple pulmonary bullae structures with a simpler surgical strategy than pulmonary cystadenomatosis. These were not included in this study. Three cases were isolated lungs, but all were intralobar types, with a surgical strategy similar to that for pulmonary cystadenomatosis, and these were included in the study. Therefore, a total of 17 cases and 18 corresponding preoperative CT scan images were included in this study. The basic information of the cases is shown in Table 2.

**Table 2:** Basic information of cases and anatomical features of lesions.

No.	Gender	Age(months)	Weight(kg)	CT Type	CT Quality	Lesion Location	Volume(cm^3^)	Volume Ratio(5%)	Trachea-real^a^	Trachea-servered^a^	Artery-real^a^	Artery-servered^a^	Vein-real^a^	Vein-servered^a^	External Boundary	Upper Boundary	Lower Boundary
1	M	86.5	27	Enhanced	High	Left lower	210	48.8	10	10 + 9-	10	10 + 9-+	10 + 9	10 + 9 + 6-	High	Low	High
2	F	4.8	7.5	Enhanced	Medium	Left lower	13.6	28.3	None	None	None	None	10	10	High	Low	High
3	M	149.6	37.6	Plain	High	Right upper	19.3	7.7	None	1	1-	1	None	1-	Low	Low	Low
4	F	86.5	22	Plain	High	Left lower	91.4	27.9	6-	6 + 8-	6-	6 + 8-	6-	6	High	High	Low
5	F	76.7	25	Plain	High	Left lower	25.6	15.5	None	None	10-, AO	10-, AO	10-	10-	Low	Low	Low
6	F	9.8	9	Enhanced	High	Right upper	23.8	39.2	None	None	None	None	None	None	High	Low	High
7	M	23.8	11.2	Plain	High	Right upper	11.2	12.4	None	None	3	3	3	3	Low	High	High
8	F	54.6	15	Plain	High	Left upper	87.9	44.8	1	1	1	1	1	1	High	High	Low
9	M	83.0	23.7	Enhanced	High	Left lower	127.5	32.5	None	None	None	None	10	10	Medium	Low	High
10	M	24.9	11.3	Enhanced	High	Left lower	63.4	40.7	None	None	10-	10	10	10	High	Low	High
11	M	72.6	20	Plain	High	Right lower	56.0	19.4	None	None	10-	10-	10-	10-	High	Low	Low
12	F	87.1	21.5	Enhanced	High	Left lower	32.1	11.7	None	None	AO	AO	6-	6-	High	Low	Low
13	M	82	19	Enhanced	High	Right lower	65.8	20.9	None	10-	10-	10-	10-	10-	High	Low	High
14	M	65.7	21	Plain	Medium	Right lower	47.5	17.7	None	None	10-	10-	10-	10-	High	Low	Low
15	F	126.9	52.5	Plain	High	Right lower	16.5	3.9	None	None	None	None	None	None	High	Low	Low
16	F	29	13	Plain	High	Left lower	29.7	28.4	None	10-	10-	10-	10-	10-	High	Low	High
17	M	74.3	21.5	Plain	High	Right lower	29.6	12.7	None	None	10-	10-	10-	10-	High	Low	High
18	F	102.1	29	Plain	High	Right upper	63.1	21.1	None	None	1-	1 + 2-	1-	1-	High	Medium	Low

^a^ Numbers 1–10 correspond to the following lung segments: apex, posterior, anterior, outer (upper lobe), inner (lower lobe), dorsal, inner basal, anterior basal, outer basal, posterior basal. “-” indicates subsegment branches. AO: Aortic-origin collateral arteries.

### 3.2 Feasibility of reconstructing lung airways, vascular branches, and CPAM lesions using CT imaging

Among 25 preoperative CT scans, 5 cases were excluded due to low-quality ratings, leaving 80% of the cases with high or medium CT image quality. Among the enrolled cases, there were 9 males (47.4%), with an average age of 68.9 ± 38.9 months and a weight of 21.2 kg (range: 13.5–24.7 kg). All cases completed 3D reconstruction of the lung lobes, trachea, vessels, and lesions, with the reconstruction time for enhanced CT images being shorter than that for plain CT images. The trachea and blood vessels on the lesion side were distinguished by different colors according to the lung segments (Figure 1).

### 3.3 CT-based analysis of the anatomical characteristics of lesions

**Figure 3:**
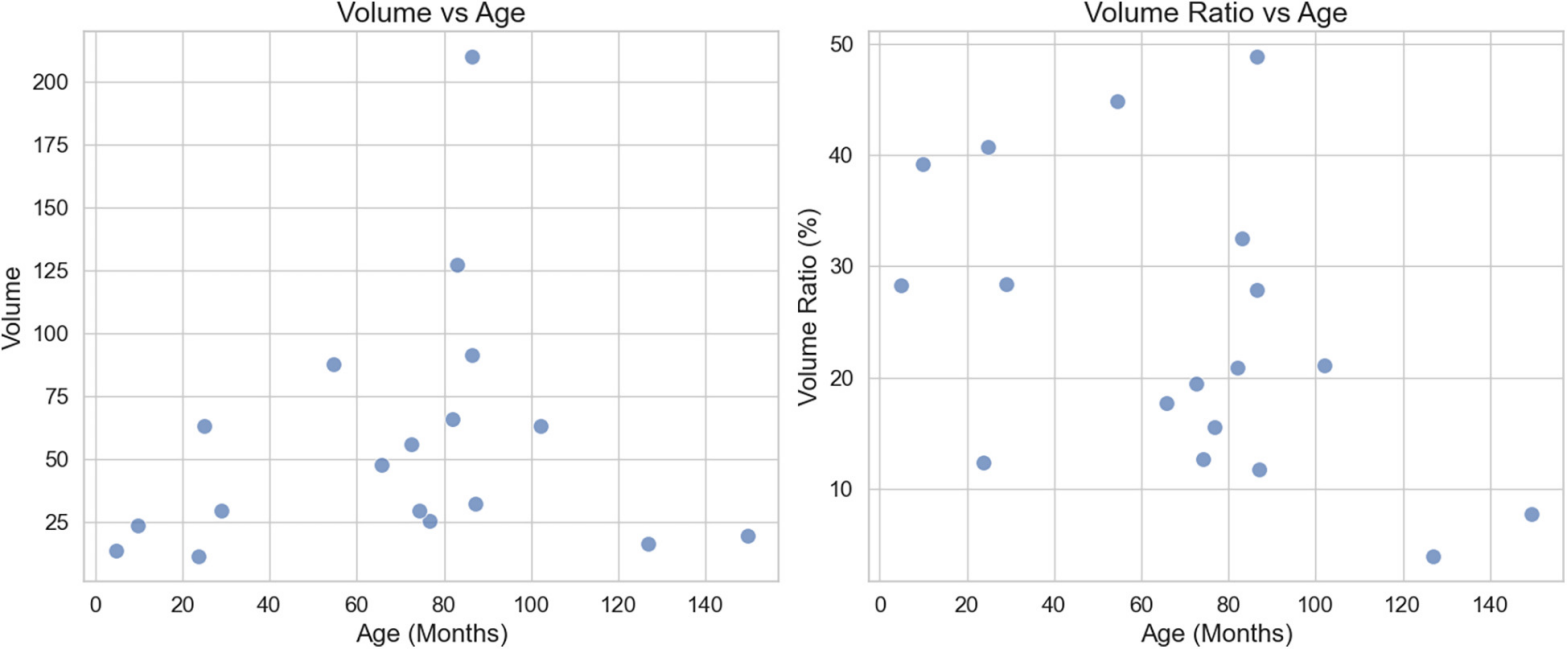
Scatter plot of lesion volume, volume ratio, and age: left: there is no significant correlation between lesion volume and age, with a correlation coefficient of *r* = 0.25. Right: There is a weak negative correlation between lesion volume ratio and age, with a correlation coefficient of *r* = −0.48.

Spatial positioning and volume: The position of the lesion was defined by the physical location within the lung segment in the affected lung lobe. The lesion distribution was as follows: 8 cases in the left lower lung (44.4%), 4 cases in the right upper lung (22.2%), 5 cases in the right lower lung (27.8%), and 1 case in the left upper lung (5.5%). The lesion volume was 47.5 cm^3^ (range: 25.6–91.4 cm^3^), and the lesion occupied 26.6 ± 12.7% of the affected lung lobe. There was no significant correlation between lesion volume and age (r = 0.25), and a weak negative correlation between the volume ratio of the lesion to the lung lobe and age (*r* = −0.48) (Figure 3).

Tracheal and vascular branches: The proportion of lesions with real supplying tracheal branches, arterial branches, and venous branches was 16.7%, 77.8%, and 83.3%, respectively. The proportion of lesions requiring severance of tracheal branches, arterial branches, and venous branches during resection was 33.3%, 77.8%, and 88.9%, respectively. The proportion of lesions requiring severance of additional tracheal branches, arterial branches, and venous branches was 27.8%, 16.7%, and 5.5%, respectively. The proportion of lesions requiring severance of first-order segmental branches of the trachea, arteries, and veins was 22.2%, 38.9%, and 38.9%, respectively. The proportion of lesions requiring severance of branches involving two lung segments for the trachea, arteries, and veins was 11.1%, 16.7%, and 5.5%, respectively.

Lesion External Boundary: In terms of the exposure of the external boundaries of the lesion, the highest exposure was seen for the external boundary, with the proportions of high, medium, and low exposure being 77.8%, 5.5%, and 16.7%, respectively. For the upper boundary, the proportions were 16.7%, 5.5%, and 77.8% for high, medium, and low exposure, respectively. For the lower boundary, the proportions were 50%, 0%, and 50% for high, medium, and low exposure, respectively.

## 4 Discussion

As mentioned in the background section, the treatment of congenital pulmonary airway malformations (CPAM) in children and the associated controversies have been discussed. Children with CPAM typically undergo chest CT scans preoperatively to assess the location, size, tracheal and vascular branching, possible inflammation, and potential pathological nature of the lesions (26, 30, 32–34). However, there are few large-scale reports involving 3D reconstruction and systematic analysis of the tracheal and vascular branches of CPAM lesions, with such studies mainly appearing in case reports (35). This study utilized medical imaging software and preoperative CT images to perform 3D reconstruction and analyze the anatomical features of CPAM lesions. It is hoped that this approach will assist surgeons in fully identifying the size, boundaries, and anatomical characteristics of the tracheal and vascular branches of lesions preoperatively, thus improving surgical outcomes.

In this group of cases, lesions were most commonly located in the lower lung, accounting for 72.2%. The lesions occupied 26.6 ± 12.7% of the volume of the affected lung lobe, with a weak correlation to age (*r* = −0.48). There may be a trend of decreasing volume ratio in older children, but larger sample sizes are needed for further validation.

From the perspective of CT image reconstruction, the proportion of lesions with real tracheal branch supply was very low, at 16.7%. One possible reason is that CPAM lesions themselves rarely have first-order segmental tracheal branches entering them, as these are generally visible on preoperative CT images or during surgery. In our study, only 2 cases (11.1%) showed tracheal branches entering the lesion's parenchyma. Another possible reason is that the study population consists of children, who have smaller tracheal diameters. Furthermore, due to factors such as respiratory artifacts, expansion compression of the lesion, and exudates within the lesion, subsegmental tracheal branches may be difficult to visualize on CT images, even though they may exist. These subsegmental tracheal branches, which may not be accurately ligated and severed, could be a major cause of bronchopleural fistulas after lesion resection. Therefore, improving cauterization of the surgical wound or suturing of residual wound edges may further reduce the incidence of postoperative pneumothorax.

CPAM differs from pulmonary bullae in that it generally involves arterial and venous branches supplying the lesion. In about 80% of the lesions in this study, arterial and venous branches were observed to supply the lesion. The real supplying vascular branches and the branches that may need to be severed during resection were not entirely consistent in some cases. Based on our image reconstruction method, 16.7% of arterial branches required severance of additional branches, whereas only 5.5% of venous branches required severance. Anatomically, arterial branches are situated between the lesion's parenchyma and venous branches. Therefore, using the venous branch plane as the boundary marker for lesion resection ensures that the majority of lesions are completely excised at the pulmonary vein branch plane. Strengthening the identification of branches entering the lesion's parenchyma and adjacent pulmonary vein branches during preoperative and intraoperative procedures, and severing the corresponding nearby branches when necessary, can further guarantee complete resection of the lesion. However, this approach also carries a potential risk: the retained adjacent lung tissue may lose its original supply of tracheal and vascular branches, as these are severed during the expanded resection. Whether these lung tissues will experience normal structural but dysfunctional conditions postoperatively requires longer-term follow-up and more detailed evaluation.

During surgery, with the help of single-lung ventilation, curving small blood vessels and cystic expansions are often observed on the lesion surface, thus distinguishing the lesion from collapsed normal lung tissue. For lesion resection, the distinction between the lesion and normal lung tissue serves as an important criterion for determining the resection range. In this study, based on CT image reconstruction, the external boundary exposure of the lesion was best, which is consistent with what was observed during direct intraoperative visualization. The external boundary of the lesion, reconstructed from CT images, can be further processed by image reconstruction methods, such as the Growth function in the Margin module of 3D Slicer software, to enhance the exposure of the boundary. This can help with intraoperative recognition of the lesion's boundary.

Based on the above results and analysis, we believe that preoperative 3D CT reconstruction can provide a more comprehensive assessment of CPAM lesion anatomy. The method proposed in this study is based on surgeons' correct understanding of lung anatomy, accurate interpretation of chest CT images, and proficiency in using the free open-source software 3D Slicer. The first two requirements are inherent skills expected of surgeons, while lung vessel segmentation using 3D Slicer can be quickly mastered by referring to online tutorials and videos. Unlike relying on simple 3D reconstruction results provided by radiology departments, this method enables surgeons to create more precise, interactive, and flexible 3D reconstructions of lesions and analyze their anatomical characteristics. It even allows for surgical procedure simulations (Video 1).This allows for the identification and comparison of the actual tracheal and vascular branches associated with CPAM lesions and those that may require transection during surgical resection. The differences between these structures could help surgeons determine the appropriate extent of resection. By constructing a virtual resection plane and carefully analyzing the distribution of tracheal and vascular branches on both the lesion side and the pulmonary hilum side, surgeons may be able to achieve more precise localization and excision of the lesion.

The purpose of this study is twofold. First, we propose a new method that uses preoperative CT imaging and 3D reconstruction to comprehensively analyze the anatomical characteristics of CPAM lesions, which may serve as a reference for surgical decision-making. Second, this method may help reduce surgical difficulty and improve surgical outcomes. The main limitations of this study include: 1. Small sample size. However, the reconstructed results cannot fully reflect the overall anatomical characteristics of CPAM. 2. The manual reconstruction of images using medical imaging software, which is time-consuming and labor-intensive. The reconstruction results may have certain quality biases due to the software capabilities and the operator's influence. 3. Our experience with CPAM lesion resection is limited, and this study is a retrospective analysis of surgical case imaging, presenting only a new approach without prospective research or further clinical validation. The anatomical features of the lesion based on image analysis may not be fully reproducible during surgery.

## 5 Conclusion

By utilizing preoperative CT imaging and corresponding imaging software, a comprehensive analysis of the anatomical characteristics of congenital pulmonary airway malformation lesions can be conducted. This may help improve the understanding of CPAM and enhance the effectiveness of lesion resection surgeries.

## Data Availability

The original contributions presented in the study are included in the article/Supplementary Material, further inquiries can be directed to the corresponding authors.
